# Traditional Chinese Medicine-derived formulations and extracts modulating the PI3K/AKT pathway in Alzheimer’s disease

**DOI:** 10.3389/fphar.2025.1528919

**Published:** 2025-03-17

**Authors:** Lan Ma, Jing Wang, Rong Zhou, Miao Chen, Zuxiu Huang, Shuyang Lin

**Affiliations:** ^1^ Department of Neurology, Wenzhou Traditional Chinese Medicine (TCM) Hospital of Zhejiang Chinese Medical University, Wenzhou, Zhejiang, China; ^2^ Department of Cardiology, Nanning Hospital of Traditional Chinese Medicine, Nanning, Guangxi, China

**Keywords:** Alzheimer’s disease, PI3K/Akt signal pathway, glycogen synthase kinase-3β (GSK-3β), amyloid-β, Traditional Chinese medicine (TCM)

## Abstract

Alzheimer’s disease (AD) is a common neurodegenerative disorder characterized by memory decline, cognitive impairment, and behavioral abnormalities. Pathologically, AD is marked by neurofibrillary tangles caused by excessive phosphorylation of Tau protein and abnormal deposition of β-amyloid (Aβ) in the brain. The PI3K/AKT signaling pathway plays a crucial role in the development, survival, and metabolic regulation of the central nervous system, particularly in neuronal growth, differentiation, and apoptosis. However, this pathway is often inhibited in AD patients.In recent years, studies have shown that herbal formulations and extracts derived from Traditional Chinese Medicine (TCM) can regulate the PI3K/AKT signaling pathway, thereby improving AD pathological models. This study reviews fundamental research on both active metabolites and compound formulations from TCM for the treatment of AD, targeting the PI3K/AKT signaling pathway.Keywords include “Alzheimer’s disease” “AD” “dementia” “PI3K” “AKT” “Traditional Chinese Medicine” “Chinese herbology” “Chinese medicine” and “TCM”.The study is based on relevant literature published over the past 15 years, primarily sourced from electronic databases such as Web of Science, PubMed, CNKI, Wanfang, and VIP databases.The findings indicate that herbal formulations and extracts derived from TCM can mitigate AD pathology by regulating the PI3K/AKT signaling pathway, reducing Tau protein phosphorylation and Aβ deposition, inhibiting inflammatory responses and oxidative stress, and alleviating neuronal apoptosis. This study enhances our understanding of the anti-AD mechanisms of TCM through the PI3K/AKT pathway and offers new insights for the future.

## Graphical Abstract

**Figure:**
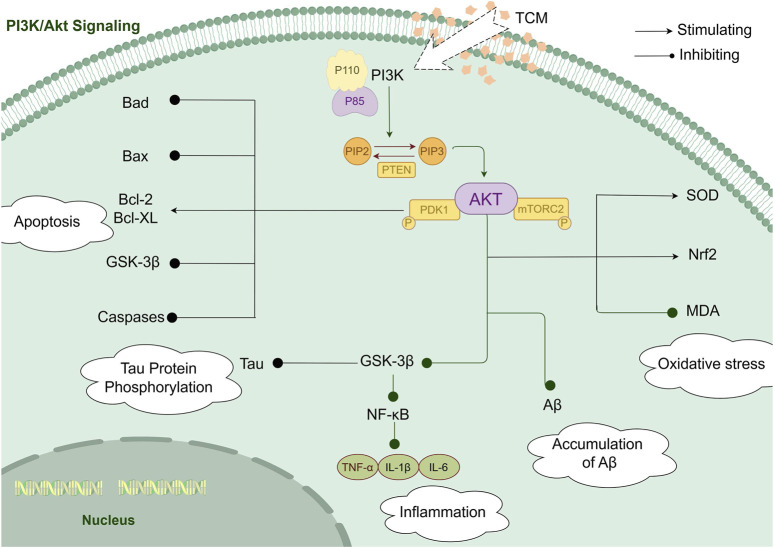
The diagram illustrates how herbal formulations and extracts derived from TCM exert potential effects on AD through modulation of the PI3K/AKT signaling pathway. Various bioactive compounds present in these formulations and extracts have been reported to influence multiple pathological mechanisms associated with AD, including cell apoptosis, Tau protein phosphorylation, oxidative stress, inflammatory responses, and Aβ deposition.While these findings suggest a mechanistic basis for the potential effects of TCM-derived formulations and extracts in AD, further well-designed pharmacological and clinical studies are required to confirm their efficacy and elucidate their precise molecular targets.

## 1 Introduction

**FIGURE 1:**
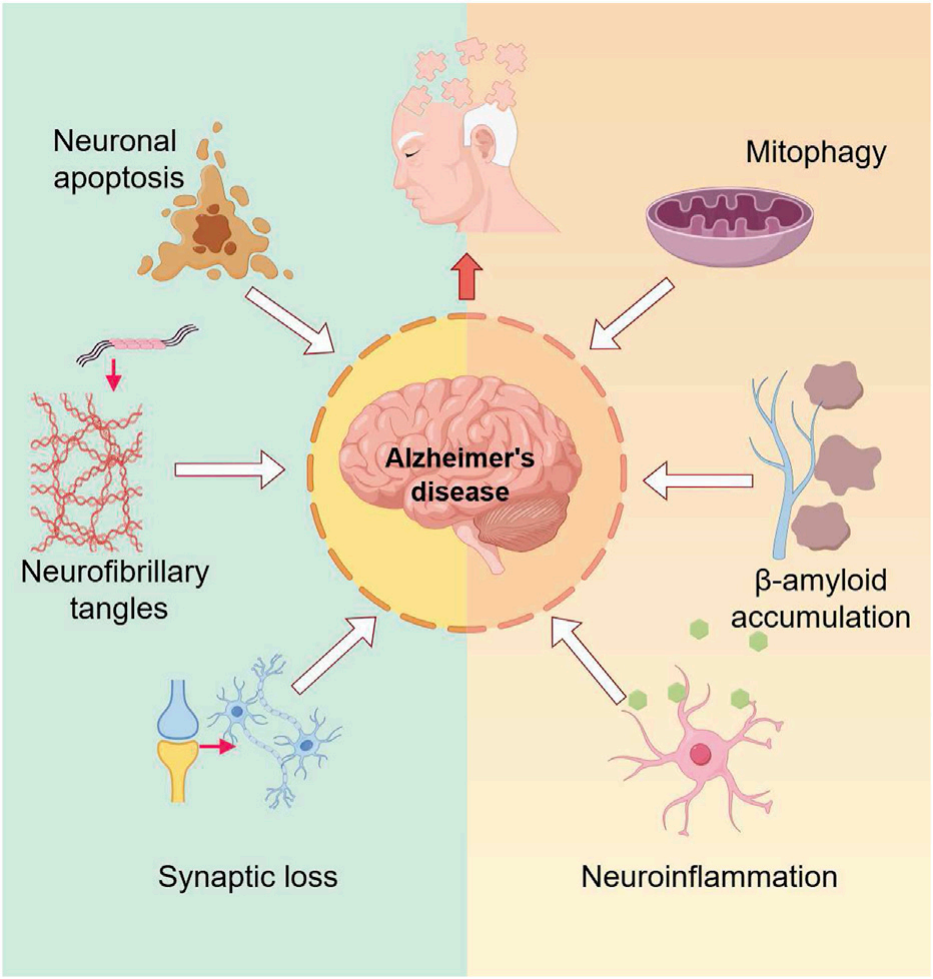
The pathogenesis of Alzheimer’s disease. The diagram illustrates the multifactorial pathogenic mechanisms of Alzheimer’s disease, including neuronal apoptosis, neurofibrillary tangle formation due to excessive tau protein phosphorylation, Aβ deposition, synaptic loss, neuroinflammation, and mitophagy.

Alzheimer’s disease (AD) is a neurodegenerative disorder characterized by an insidious onset and progressive cognitive impairment. With the aging population in China, even in the world, the incidence of AD continues to rise. Currently, there are 15.07 million dementia patients aged 60 and above in China, of which 9.83 million suffer from AD (R et al., 2022). AD has become a major medical and social issue. The neuropathological hallmarks of AD include extracellular deposits of amyloid-β (Aβ) plaques and intracellular neurofibrillary tangles (NFT) composed of aggregated and hyperphosphorylated Tau protein (AuthorAnonymous, 2023). The pathogenesis of Alzheimer’s disease is shown in Figure 1. Various signaling pathways are involved in the pathological processes of AD, with the phosphoinositide 3-kinase/protein kinase B (PI3K/AKT) signaling pathway playing a critical role in the central nervous system, including functions such as cell survival, autophagy, neurogenesis, neuronal proliferation and differentiation, and synaptic plasticity. It is especially closely related to AD pathological processes like Tau protein phosphorylation and apoptosis, making it a key pathway in AD treatment (Manish Kumar and Bansal, 2022). Multiple studies have shown that activation of the PI3K/AKT signaling pathway has a positive effect on AD treatment.

The activation of the PI3K/AKT pathway begins with the binding of insulin to the insulin receptor (IR). Its numerous downstream targets, including AKT, glycogen synthase kinase-3β (GSK-3β), endothelial nitric oxide synthase (eNOS), mammalian target of rapamycin (mTOR), and Bad, play roles in promoting cell survival, proliferation, growth, and metabolic pathway changes. This pathway serves as a critical drug target for various AD-related pathogenic factors, including aging, abnormal glucose metabolism, Aβ deposition, synaptic dysfunction, and neuronal apoptosis (Seidler and Barrow, 2022; Zhang et al., 2021). Activation of this pathway can help alleviate oxidative stress and inflammatory responses, reduce Aβ aggregation and NFT formation, and block the pathogenesis of AD. A growing number of natural products, as well as synthetic and semi-synthetic molecules, have been found to mitigate AD pathology by modulating the PI3K/AKT pathway (Kumar and Bansal, 2022; Huang et al., 2018).

In recent years, an increasing number of studies have suggested that certain herbal formulations and extracts derived from Traditional Chinese Medicine (TCM) may influence the pathological processes of Alzheimer’s disease (AD) by modulating the PI3K/AKT signaling pathway. These studies have reported that some bioactive compounds exhibit potential anti-inflammatory and antioxidant effects, inhibit apoptosis, and alleviate oxidative stress. However, the precise molecular mechanisms underlying these effects require further investigation.A deeper exploration of how TCM-derived active compounds or extracts regulate AD-related pathological changes through the PI3K/AKT pathway could contribute to a better understanding of their potential mechanisms and provide new research directions for AD treatment. This review encompasses experimental research on TCM-related formulations and extracts in regulating AD via the PI3K/AKT pathway. It explores the proposed mechanisms, assesses available evidence on their efficacy, and discusses future research directions.”

This review used the Web of Science, PubMed, CNKI, Wanfang, and VIP databases as data sources. The keywords included “Alzheimer’s disease” “AD” “dementia” “PI3K” “AKT” “Traditional Chinese Medicine” “Chinese herbology” “Chinese medicine” and “TCM” The search period was from January 2010 to January 2025.

## 2 Alleviation of apoptosis

Apoptosis is a cell death mechanism that regulates neuronal development, characterized by DNA fragmentation and the loss of mitochondrial membrane integrity (Gupta et al., 2023; Fleisher, 1997). It has been reported that neuronal loss in AD exhibits characteristics of apoptosis, pyroptosis (programmed necrosis), or necroptosis. Extensive neuronal loss, attributed to apoptosis, is closely related to the progression of AD (Sharma et al., 2021). The degree of neuronal loss worsens with the severity and duration of the disease. The extrinsic and intrinsic pathways are the main executors of apoptosis in mammalian cells, with caspases being the primary enzymes involved. The PI3K/AKT signaling pathway regulates apoptosis by modulating caspase-3 activity (VK et al., 2021). Activation of the PI3K/AKT pathway can counteract neuronal apoptosis (Khezri et al., 2023). Activated AKT promotes the phosphorylation of Bad at the serine 136 site. When Bad is poorly phosphorylated, it translocates to the mitochondrial outer membrane, inactivating anti-apoptotic Bcl-2 family proteins like Bcl-2 and Bcl-XL, thereby triggering mitochondrial-dependent apoptosis (Zhang et al., 2021; Cheng et al., 2020). Glycogen synthase kinase-3β (GSK-3β) is also involved in activating caspase-2 and caspase-8, which can induce the cleavage of Bid (Bcl-2 homology three interacting domain death agonist) and the release of cytochrome C, leading to mitochondrial dysfunction and apoptosis (Lin et al., 2016; Kumari et al., 2023). Furthermore, GSK-3β can promote the mitochondrial apoptotic pathway by increasing the Bax/Bcl-2 ratio, contributing to neurodegenerative diseases (Toral-Rios et al., 2020).

Studies have reported that Shenqi Pill may help mitigate learning and memory impairments, pathological damage, and cell apoptosis in Alzheimer’s disease (AD) rat models. This effects are associated with increased expression of Bcl-2, PI3K, PDK1, P-AKT, and GSK-3β, along with decreased phosphorylation levels of Bax and Caspase-3 (Huang JHJ. et al., 2022). Similarly, Dabu Yuanjian has been observed to enhance cognitive function in AD rat models, potentially by upregulating Bcl-2, P-AKT, and P-GSK-3β, while decreasing Caspase-3 expression. Transmission electron microscopy findings suggest treatment with Dabu Yuanjian may alleviate myelin-like changes and mitochondrial swelling in the hippocampus of AD rat models (Xi et al., 2022). Modulated Shuyu Pill-containing serum has been found to a reduction in the levels of the phosphorylated α-subunit of eukaryotic translation initiation factor 2 (p-eIF2α/eIF2α) in the apoptosis-related pathway in primary neurons of APP/PS1 mice, effectively increased the expression of P-AKT/AKT and Nrf2, and mitigating endoplasmic reticulum stress-induced neuronal apoptosis (Jing et al., 2019). Metabolites Danshen, composed of Danshen, Panax notoginseng, and Borneol, has been reported to inhibit Bad expression and increase P-AKT expression, providing protective effects for hippocampal neurons in AD mouse models (Liang et al., 2018).Polygala saponins, active metabolites of Polygala, have also been investigated. Junping Wang and colleagues found that Polygala saponins combined with β-asarone enhanced cell viability in AD cell model, increased AKT expression, inhibited GSK-3β activation, and reduced apoptosis rates (Junping et al., 2018). Forsythoside A, a phenylethanoid glycoside isolated from the dried fruit of Forsythia (Qu et al., 2012), was shown by Chunyue Wang and colleagues to improve cell viability in AD models, reduce apoptosis rates, and downregulate caspase-3, -8, and -9 levels (Wang H. C. et al., 2020).Aconitine, a key active component of Aconitum, has pharmacological effects such as cardiotonic, analgesic, antitumor, and immune-modulatory properties (Xiu et al., 2019). Weizhi Quan and colleagues observed that aconitine could reduce apoptosis rates and GSK-3β expression in AD cell models (Zhi-quan et al., 2021). Hydroxy-α-sanshool, the main active component of Sichuan pepper, is the most abundant amide metabolite in the plant and has been shown to improve learning and memory (Khoshsirat et al., 2019). Ruolan Li and colleagues found that hydroxy-α-sanshool increased the expression of P-AKT, P-PI3K, AKT, and Bcl-2 in AD cell models, while reducing the expression of caspase-3 and Bax, thus alleviating apoptosis (Lan, 2021).Lastly, crocetin has been observed to significantly increase the expression levels of PI3K, Akt, and Bcl-2 proteins and mRNA in hippocampal neuron models of AD induced by Aβ_25-35_, while decreasing the expression of Bax protein and mRNA, thereby inhibiting apoptosis and protecting hippocampal neurons (Yan Y. et al., 2020).

## 3 Inhibition of tau protein phosphorylation

Tau protein is a microtubule-associated protein widely expressed in the nervous system. Under pathological conditions, abnormal post-translational modifications of Tau, primarily hyperphosphorylation, reduce its ability to bind to microtubules. This leads to a conformational change in Tau from its natural unfolded state to paired helical filaments and neurofibrillary tangles (NFTs) (Ossenkoppele et al., 2022; Chu et al., 2024). NFTs are a hallmark and definitive evidence of Alzheimer’s disease (AD) pathology, and their presence is positively correlated with cognitive impairment (Ashton et al., 2021; Naseri et al., 2019). Dysregulation of the PI3K/AKT signaling pathway is a major factor in Tau hyperphosphorylation. Overexpression of AKT can significantly reverse Aβ-induced Tau phosphorylation, while excessive activation of GSK-3β is also closely related to Tau hyperphosphorylation (Shi et al., 2019; Wang et al., 2013). Dysregulation of PI3K/AKT signaling leads to increased GSK-3β activity and decreased PP2A activity, which contributes to tau hyperphosphorylation and the formation of NFTs (Yang et al., 2014).GSK-3β can phosphorylate Tau at multiple residue sites. Studies have shown that after transfecting GSK-3β into rat brains, increased GSK-3β expression and abnormal Tau phosphorylation were detected, with Tau hyperphosphorylation co-localizing with GSK-3β, suggesting that GSK-3β-induced Tau hyperphosphorylation is involved in the mechanism of neurodegenerative diseases (Wang et al., 2013). Autopsies of AD patients revealed reduced PI3K/AKT signaling activity in the frontal cortex, along with elevated levels of phosphorylated GSK-3β and abnormally hyperphosphorylated Tau (Ochiai et al., 2021).

Studies have demonstrated that the TCM formula Jinsiwei(GAPT) exhibited the ability to improve learning and memory abilities in APP/PS1 Alzheimer’s disease (AD) mouse models by reducing the expression of phosphorylated Tau (P-Tau) and increasing the expression of P-AKT and P-AKT/AKT (Li, 2017; Zhuo, 2022). Yuanzhi Powder has been shown to decrease the expression of P-Tau (Ser199)/Tau5 and P-Tau (Thr231)/Tau5 in the hippocampus of AD rat models, while increasing the expression of P-AKT/AKT and P-GSK-3β/GSK-3β (Peijun et al., 2020). Xiaoyao Powder decreases GSK-3β expression in AD mouse models, thereby inhibiting Tau hyperphosphorylation (Wei-Xian et al., 2014).Litchi seed polyphenols, derived from litchi seeds, have been reported by Rui Xiong et al. to inhibit the expression of P-IRS-1 (Ser612) in AD cell models, restore the expression of P-PI3K (Tyr199/Tyr458), P-AKT (Thr308), and P-GSK-3β (Ser9), and ultimately suppress Tau hyperphosphorylation (Xiong et al., 2020). Curcuma aromatica volatile oil, an active component from the dried roots of *Curcuma aromatica*, significantly reduces the phosphorylation levels of Tau protein (Thr231, Ser404) in AD mouse models, while increasing the expression of P-PI3K/PI3K and P-AKT/AKT, as shown by Qi Yue et al. (Yue et al., 2017).Paeoniflorin, the main bioactive component of *Paeonia* and a monoterpene glycoside, has beneficial effects on neurodegenerative diseases (Chao-fang et al., 2023). Research by Xiao-Hui Ma showed that paeoniflorin improves morphological changes in AD cell models, such as reducing cell swelling and synaptic shrinkage, this is achieved by lowering Tau phosphorylation levels, increasing AKT and GSK-3β phosphorylation levels, and stabilizing microtubule structures (Ma et al., 2018). In APP/PS1 AD mouse models, Forsythoside A has been reported to enhance memory and cognitive abilities, reduces Aβ plaque deposition in the brain, and inhibits Tau protein phosphorylation (Wang C. et al., 2020).Osthole, a coumarin metabolites extracted from Cnidium monnieri, was found by Ni Yingnan and colleagues to reduce the expression of P-Tau (Ser202), effectively enhances PI3K, P-AKT/AKT, and P-GSK-3β/GSK-3β expression, and alleviate cognitive dysfunction in AD mouse models (Ying-nan et al., 2019).

## 4 Regulation of inflammatory responses

Neuroinflammation refers to the complex immune response of the central nervous system (CNS) to various endogenous or exogenous stimuli, such as misfolded proteins, toxins, and pathogens. This process leads to the infiltration of inflammatory cells, gliosis, and neuronal loss in brain tissue (Stephenson et al., 2018). Aging-induced neuroinflammatory responses play a crucial role in the onset and progression of AD (Calsolaro and Edison, 2016; Leclerc et al., 2023). Persistent neuroinflammation in the CNS causes chronic microglial activation, which releases inflammatory mediators and initiates an inflammatory response. This chronic inflammation eventually results in neuronal death and cognitive dysfunction (Wang Y. et al., 2024). Microglial activation and the inflammatory cascade mediated by pro-inflammatory factors are fundamental mechanisms in AD pathogenesis (Wang et al., 2022a). Pro-inflammatory cytokines, such as tumor necrosis factor-α (TNF-α), interleukin-1β (IL-1β), and interleukin-6 (IL-6), play a central role in AD. Studies have shown that the levels of TNF-α are significantly elevated in the plasma and cerebrospinal fluid of AD patients (Decourt et al., 2016).In animal models of brain diseases, including AD, upregulation of the PI3K/AKT signaling pathway can inhibit downstream targets such as nuclear factor kappa-B (NF-κB) (Yang et al., 2020), which leads to a reduction in the gene expression and activity of pro-inflammatory cytokines like TNF-α (Bathina et al., 2020). Conversely, the crosstalk between GSK-3β and NF-κB can increase the expression of pro-inflammatory chemokines and apoptotic factors, ultimately causing severe neurodegeneration. This interplay between inflammatory pathways and PI3K/AKT signaling highlights the critical role of neuroinflammation in AD pathology and provides a therapeutic target for modulating inflammation in AD treatment (Shih et al., 2015).

Schisandrin and nootkatone, active metabolites of Schisandra chinensis and Alpinia oxyphylla, respectively, have been shown to significantly increase the expression of P-PI3K/PI3K, P-AKT/AKT, and P-GSK-3β/GSK-3β in Aβ1-42-induced AD cell models. These metabolites also reduce the expression of inflammation-related proteins, such as NF-κB, inhibitor of kappa B kinase (IKK), IL-1β, IL-6, and TNF-α (Qi et al., 2020). Tanshinone, a primary active metabolite in Salvia miltiorrhiza (derived from Danshen), has been found to lower the levels of IL-6, TNF-α, and P-NF-κB/NF-κB in the brain tissue of AD mouse model, while increasing the expression of BDNF, P-PI3K/PI3K, and P-AKT/AKT, thereby improving cognitive function (Yi et al., 2021). Gardenia has been shown to effectively lower the expression of pro-inflammatory factors TNF-α and IL-1β in the 3×Tg-AD mouse model (Meng, 2018). Additionally, the modified formula San Jia San is capable of alleviating neuroinflammation in AD cell models by downregulating PI3K, P-AKT, IL-1β, IL-6, and TNF-α, while upregulating anti-inflammatory cytokines IL-4 and IL-10 (Miao, 2018).

## 5 Relieve of oxidative stress

Oxidative stress is the result of an imbalance between pro-oxidants and antioxidants in the body, leading to mitochondrial damage and dysfunction. Mitochondrial dysfunction, in turn, causes the accumulation of reactive oxygen species (ROS) and exacerbates oxidative stress, ultimately resulting in age-related neurodegenerative diseases (Bai et al., 2022). The oxidants and oxidative products generated under oxidative stress can increase the expression of amyloid precursor protein (APP), leading to the aggregation of Aβ (Tamagno et al., 2012). Aβ itself can promote the increase of ROS and induce the occurrence of oxidative stress (Ill-Raga et al., 2010). The inhibition of the PI3K/AKT signaling pathway can trigger insulin resistance, leading to mitochondrial dysfunction and the subsequent surge of various types of free radicals. Conversely, the activation of the PI3K/AKT pathway can inhibit oxidative stress by enhancing the expression of superoxide dismutase (SOD) (Jiang et al., 2015). Research has shown that the PI3K/AKT signaling pathway enhances the antioxidant pathway of nuclear factor erythroid 2-related factor 2 (Nrf2) (Zhang et al., 2019), which is the main regulatory factor for antioxidants (Bahn and DG, 2019). Due to the impairment of the PI3K/AKT pathway, the unregulated activity of GSK-3β accelerates the biosynthesis of free radicals (Kanninen et al., 2011). The activity of GSK-3β enhances oxidative stress (Koundouros and Poulogiannis, 2018). By upregulating the expression and activity of eNOS in the PI3K/AKT pathway in the brains of rats, levels of malondialdehyde (MDA) decreased, while reduced glutathione (GSH) levels and SOD activity increased, significantly improving the memory of the rats (Xu et al., 2015).

Research conducted by Ma Tao and colleagues found that administering a decoction of Rehmannia to APPsw/PS1ΔE9 AD mouse models, the latency to enter a dark room was prolonged, the number of errors decreased, and the expression levels of SOD, GSH-PX, P-AKT, and P-GSK-3β effectively increased (Tao et al., 2014). Research by Qiu Jing and colleagues reported that the intervention of Jiajian Shuyu Pills improved the learning and memory abilities of APP/PS1 AD mouse models improved significantly, with a notable increase in the expression levels of Nrf2 in the hippocampus (Jing et al., 2019). Deer antler peptides, one of the main active metabolites of deer antlers, are composed of various amino acids and have a significant role in promoting neuronal regeneration. Studies have shown that deer antler peptides have the potential to enhance the expression levels of SOD, GSH-PX, PI3K, and AKT in AD cell models while reducing the expression levels of MDA (Xin, 2021). The Bushen Jianpi Kai Xin Decoction has also been demonstrated to significantly increase the expression of SOD in D-galactose + Aβ_1-42_ induced AD rat models, reduce the expression of NOS, and improve oxidative stress levels (Rong, 2018). These processes are inherently connected to the PI3K/AKT signaling cascade.

## 6 Reduction the accumulation of Aβ

Accumulation of Aβ is a key early pathophysiological event in AD, leading to neurodegeneration and cognitive impairment by inducing abnormal accumulation of tau protein (Walsh and DJ, 2007; Selkoe and J, 2016). Excessive accumulation of Aβ in the brain can trigger various pathological changes, including neuronal degeneration and apoptosis caused by the inactivation of AKT (Zeng et al., 2016). Aggregated Aβ induces tau hyperphosphorylation by enhancing the activity of GSK-3β and cyclin-dependent kinases (CDK-5) (Terwel et al., 2008). Multiple studies have shown that Aβ plaques downregulate several neurotrophic factors, such as brain-derived neurotrophic factor (BDNF), negatively regulating the PI3K/AKT pathway, resulting in significant cognitive impairment (Chen et al., 2009; Garabadu and J, 2019). The activation of the PI3K/AKT/GSK-3β pathway can trigger protective factors against Aβ neurotoxicity (Yin et al., 2011). The PI3K/AKT signaling pathway is involved in autophagy induced by Aβ_25-35_ and is highly correlated with Aβ clearance mediated by autophagy. Activation of PI3K/AKT can offset the neuronal effects induced by Aβ by influencing Aβ production/clearance and cell death (Sofola et al., 2012; Sánchez-Alegría et al., 2018; Sotolongo et al., 2020). GSK-3β plays an important role in the mechanism of amyloidosis in AD. GSK-3 alters Aβ levels by regulating APP processing (Phiel et al., 2003; Rockenstein et al., 2007). In the familial AD (FAD) 5 × FAD mouse model, increased expression of GSK-3 (α/β) isoforms hinders Aβ clearance in the brain, leading to increased Aβ plaque deposition and memory deficits (Avrahami et al., 2013).

Research has indicated that the Yifei Wenyang Huazhuo Decoction tends to reduce Aβ levels in the neurons of AD rat models, lowering the expression levels of PI3K and AKT (Jinping et al., 2019). Puerarin, a bioactive isoflavone glycoside extracted from Pueraria, has been observed to exert neuroprotective effects in AD through various mechanisms (Haixia et al., 2023). Mei Zhengrong and colleagues found that puerarin exhibits the ability to improve learning and memory deficits in APP/PS1 AD mouse models by reducing Aβ production and increasing P-GSK-3β expression (Zheng-rong et al., 2016). Cui-Zhu Yang and colleagues found that Astragaloside enhances cognitive function in APP/PS1 AD mouse models by activating the PI3K/AKT pathway, reducing hippocampal neuronal damage, and alleviating Aβ pathology (Yang et al., 2023). Curcumin, a lipophilic phenolic pigment extracted from the rhizome of turmeric, is the main active component of turmeric (Wei-wei et al., 2024). Wang Chen’s research found that curcumin treatment improved cognitive dysfunction in AD mouse models, reducing the number of Aβ-positive cells in the hippocampus as well as the expression of PI3K, AKT, and mTOR (Chen, 2013). The essential oil of Acorus tatarinowii, obtained from the dried rhizome of the plant, combined with total ginsenosides from ginseng, has been demonstrated to increase the expression of P-AKT in AD mouse models while decreasing the expression of Aβ_1-42_ (Zhen, 2016). Ginsenosides are one of the main active metabolites of ginseng and have protective effects on the central nervous system (Jing et al., 2023). Ginsenoside CK has shown potential in reducing the extracellular Aβ levels in AD cell models while increasing the expression of PI3K, AKT, and P-AKT (Jia-nan et al., 2021). Research findings have shown that upregulation of the PI3K/AKT pathway eliminates Aβ plaque formation in transgenic *Drosophila* models of AD (Zhang et al., 2016).

## 7 Discussions and perspective

TCM has a long-standing history in China, known for its therapeutic efficacy and minimal adverse reactions. In contrast to single-component drugs, herbal formulations and extracts derived from TCM offers the advantage of multi-component, multi-pathway, and multi-target approaches, which makes it a promising candidate for treating complex chronic diseases such as AD. Studies have identified the PI3K/AKT signaling pathway as a key regulator of neuronal cell growth and survival. Several traditional Chinese herbal formulations, including Kaixin San, Dihuang Yinzi, and Liuwei Dihuang Wan (Table 1), as well as potent TCM metabolites like Cnidii Monnieri, ginsenosides, and paeoniflorin (Table 2), have demonstrated beneficial effects in AD models. Table 3 shown the classifications, botanical drug and family of anti-AD active ingredients of Chinese botanical drug. These findings suggest that herbal formulations and extracts derived from TCM can potentially alleviate Aβ accumulation, inhibit tau hyperphosphorylation, reduce neuronal apoptosis, counteract neuroinflammation and oxidative stress, and enhance synaptic function by regulating the PI3K/AKT pathway, thus improving memory and cognitive function in AD. These results provide strong evidence supporting the effectiveness of herbal formulations and extracts derived from TCM in AD treatment and emphasize the importance of targeting the PI3K/AKT pathway. Although significant progress has been made in the basic research on TCM for the treatment of AD, the complexity of its components and the unknown, intricate changes that occur in the body make the current research results speculative. Further in-depth investigation is needed to clarify the specific components and molecular mechanisms through which TCM intervenes in AD, in order to better understand its therapeutic effects and guide clinical applications. The following are some limitations in the research design of TCM for the treatment of AD, existing issues, and future research needs.

**TABLE 1:** Traditional Chinese botanical drugs for Treating Alzheimer’s Disease by Intervening in the PI3K/AKT Pathway.

Botanical drug	Metabolites	Experiments	Animal or cell	Doserange	PosC	NegC	Duration	Model	Molecular mechanisms and outcomes
Kaixin San (1:1:50:25) (Yan et al., 2020b)	Ginseng, Polygala Poria, Acorus	Alcl3(90 mg/kg) i.g. +D-gal(180 mg/kg) i.p	KM mice	48, 24、12 g/kg	—	0.9%Nacl	4 weeks	*In vivo*	↑PI3K, P-AKT, P-GSK-3β
Dihuang Yinzi (3:3:3:3:3:3:3:3:3:3:3:3:3:2:1) (Tao et al., 2014)	Rehmannia, Cornus, Cistanche, Morinda, Aconite, Cinnamon, Ophiopogon, Dendrobium, Schisandra, Poria, Acorus, Polygala, Mint, Ginger, Jujube	—	APPsw/PS1ΔE9 mice	5, 2. 5, 1. 25 g/kg	Donepezil	0.9%Nacl	150 days	*In vivo*	↑SOD, GSH-PX, P-AKT, P-GSK-3β, Bcl-2/Bax ↓MDA
Suan Zao Ren Decoction (10:2:2:3:1) (Qinghua et al., 2020)	Sour Jujube Seed, Poria, Chuanxiong, Anemarrhena, Honey-Fried Licorice	—	APP/PS1 mice	12.96, 25.92 g/kg	Donepezil	Water	30 days	*In vivo*	↑PSD-95, SYN, P-PI3K(Tyr607), P-AKT(Ser473), P-GSK-3β(Ser9) ↓P-Tau(Ser205), P-Tau(Ser396), P-Tau(Ser404)
Yizhi Zhi Dai Fang (15:15:15:15:10:10:10:10:10:6) (ZHAO et al., 2020)	Rehmannia, Astragalus, Cardamom Seed, Deer Antler Glue, Acorus, Polygala, Curcuma, Angelica Sinensis, Chuanxion, Wine-Processed Rhubarb	Aβ_1-42_(5uL)H.I.	SD rats	1488 mg/kg	Donepezil	0.9%Nacl	28 days	*In vivo*	↑P-AKT/AKT,P-GSK-3β/GSK-3β ↓BAX
Shenqi Yizhi Granules (Lixia et al., 2018)	Astragalus, Scutellaria, Ginseng,et al	Aβ_1-42_ H.I.	SD rats	9.8, 4.9, 2.45 g/kg	Donepezil	Water	60 days	*In vivo*	↑PI3K, AKT
Bushen Jianpi Kaixin Formula (Rong, 2018)	Rehmannia, Chinese Yam, Cornus, Ginseng, Poria, Polygala, Acorus, et al	D-Gal(300 mg/kg) i.p.+ Aβ_1-42_ H.I.	Wistar rats	20.8,10.4, 5.2 g/kg	Donepezil	Water	28 days	*In vivo*	↑SOD, Ngb, PI3K, AKT ↓NOS
Yifei Wenyang Huazhuo Decoction (15:15:15:15:15:15:15:15:15:10:6) (Jinping et al., 2019)	Processed Aconite, Epimedium, Raw Sun-Dried Ginseng, Dried Ginger, Morinda, Cinnamon Twig, Pinellia, Acorus, Notoginseng, Platycodon, Rhubarb	Aβ_1-40_(1uL) H.I.	SD rats	1.25, 2.5, 5 g/kg	Naofukang Capsules	0.9%Nacl	4 weeks	*In vivo*	↑mTOR ↓PI3K, AKT, Beclin1, LC3
Yuan Zhi San (4:6:5:3:4) (Peijun et al., 2020)	Polygala, Acorus, White Poria, Ginseng, Coptis	Aβ_1-40_(5uL) H.I.	SD rats	3, 6, 12 g/kg	Donepezil	Water	—	*In vivo*	↑P-AKT/AKT, P-GSK-3β/GSK-3β ↓P-Tau(Ser199)/Tau5, P-Tau(Thr231)/Tau5
Jiajian Shuyu Wan (15:12:12:10:9:9:10:10:5:6:6:7:9:5) (Jing et al., 2019)	Chinese Yam, Fo-ti, Rehmannia, Codonopsis, Atractylodes, Poria, White Peony, Angelica Sinensis, Chuanxiong, Eucommia, Polygala, Acorus, Goji Berry, Schisandra	— —	APP/PS1 mice Primary Neurons from APP/PS1 mice	14 g/kg 5%, drug-containing serum	— —	0.9%Nacl 10%drug-free serum	28 days 12 h	*In vivo* *In vitro*	↑P-AKT/AKT, Nrf2 ↓GSK-3β
Liu Wei Di Huang Decoction (8:4:4:3:3:3) (Kunpeng et al., 2016)	Rehmannia, Dried Chinese Yam, Cornus, Alisma, Poria, Paeonia	D-gal(500 mg/kg) s.c	KM mice	2, 1, 0.5 g/kg	VitE	Water	8 weeks	*In vivo*	↑Wnt3a ↓GSK-3β
Xiao Yao San (10:10:10:10:5:1:1) (Wei-Xian et al., 2014)	Bupleurum, Angelica Sinensis, White Peony, Poria, Honey-Fried Licorice, Fresh Ginger, Peppermint	Aβ_1-42_(2uL)H.I. +D-gal(100 mg/kg) i.p	SD rats	10 g/kg	Oxiracetam	0.9%Nacl	28 days	*In vivo*	↓GSK-3β
Wen Pi Tong Luo Kai Qiao Decoction (3:1:1:1:1:1) (Jinping et al., 2013)	Astragalus, Euryale Seed, Notoginseng, Acorus, Fo-ti, Gynostemma	OA(1.5uL) H.I.	SD rats	4.15, 8.3, 16.9 g/kg	Huperzine-A	Water	21 days	*In vivo*	↓GSK-3β
Gardenia (Meng, 2018)	— —	— —	3×Tg mice 3×Tg mice Hippocampal neurons	100 mg/kg 10uM	\ \	Water culture medium	8 weeks 24 h	*In vivo* *In vitro*	↑Bcl-2, PP2A ↓Aβ_1-42_, P-Tau(Ser396),GSK-3β, APP, Bax, BACE1, TNF-α, IL-1β
Da Bu Yuan Jian (2:3:1:1:3:2:2:3) (Xi et al., 2022)	Ginseng, Eucommia, Chinese Yam, Cornus, Honey-Fried Licorice, Rehmannia, Angelica Sinensis, Goji Berry	Aβ_25-35_(4uL)H.I.	SD rats	5.31 g/kg	—	Water	28 days	*In vivo*	↑Bcl-2, P-AKT, P-GSK-3β ↓caspase-3
Wuhe Xuduan (Jie, 2023)	—	WT + GFX(5uL) H.I.	SD rats	0.07, 0.14, 0.28 g/kg	—	Water	2 and 4 weeks	*In vivo*	↑PI3K,P-AKT, P-GSK-3β (Ser9) ↓GSK-3β, P-Tau(Ser396), P-Tau(Ser262)
Acorus (Juan, 2022)	—	D-gal(0.43.74 mg) s.c	C57BL/cnc mice	1, 2, 4 g/kg	Donepezil	0.9%Nacl	13 weeks	*In vivo*	↑P-PI3K, P DGFR-β, LRP-1 ↓BACE1
Shen Hui Decoction (20:7:4:10:10:6:6:1:4) (Lexuan, 2022)	Rehmannia, Cornus, Polygala, Ziziphus Seed, Biota Seed, Poria Spirit, Ginseng, Acorus, White Mustard Seed	AlCl3(100ug/L) soak	Zebrafish	0.6 mg/mL	Donepezil	Water	14 days	*In vivo*	↑PI3K, AKT, P-AKT, mTOR ↓APP, Aβ_1-42_
Zi Shen Xing Nao Decoction (24:24:12:9:15:12:20:10:20:20:10:10) (Wang et al., 2022b)	Rehmannia Root, Raw, Rehmannia Root, Prepared, Cornelian Cherry Fruit, Processed Pinellia Rhizome, Acorus Tatarinowii Rhizome, Szechuan Lovage Rhizome, Salvia Miltiorrhiza Root, Angelica Sinensis Root, Poria, Rhodiola Root, Bamboo Sap, Earthworm	Aβ_25-35_(1uL) H.I.	SD rats	16.74 g/kg	Donepezil	Water	4 weeks	*In vivo*	↑BDNF, TrkB, PI3K, AKT
Zhi Nao Jiao Nang (15:15:15:12:10:8:10:8) (Na, 2022)	Codonopsis Root, Cistanche, Astragalus Root, Polygonatum Rhizome, Curcuma Root, Acorus Tatarinowii Rhizome, Szechuan Lovage Rhizome, Earthworm	—	APP/PS1 mice	3.5, 7, 14 g/kg	Donepezil	0.9%Nacl	28 days	*In vivo*	↑PI3K, P-AKT ↓P-GSK-3β
Liu Wei Di Huang Wan (8:4:4:3:3:3) (Xinyun, 2022)	Rehmannia Root, Prepared, Chinese Yam, Cornelian Cherry Fruit, Moutan Cortex, Poria, Alisma Rhizome	—	SAMP8	2.70, 1.350 g/kg	Donepezil	0.9%Nacl	2 months	*In vivo*	↑IR-β, P-IRS-1, P-PI3K, P-AKT ↓GSK-3β
Gui Ling Ji (Fengrui, 2022)	Ginseng, Deer Antler, Sea Horse, Sparrow Brain, Prepared Rehmannia Root, Goji Berry, Achyranthes Root, Licorice Root, Isatis Leaf	—	APP/PS1 mice	0.75, 1.5, 3 mg/d	—	Water	8 months	*In vivo*	↑PI3K ↓GFAP
Shen Zhi Ling oral liquid (Gaofeng, 2021)	Codonopsis Root, Cinnamon Twig, White Peony Root, Honey-Fried Licorice Root, Poria, Dried Ginger, Processed Polygala Root, Acorus Tatarinowii Rhizome, Dragon Bone, Oyster Shell	STZ(3 mg/kg) H.I.	C57/BL6J mice	50, 25, 12.5 g/kg	Donepezil	0.5%CMC	3 months	*In vivo*	↑IRS2, PI3K, P-PI3K, AKT, P-GSK-3β, GLUT3, P-AKT, GLUT1 ↓GSK-3β
Schisandra-Prepared Rehmannia (Yubao, 2018)	—	Aβ_1-42_(5uL)H.I + D-gal(150 mg/kg) s.c	SD rats	0.8, 1.6, 3.2 g/kg	Huperzine A	0.9%Nacl	28 days	*In* *vivo*	↑BDNF, TrkB, CREB, SYP, PSD-95, AKT ↓Aβ_1-42_, Aβ_1-40_, GSK-3β, Tau, APP
Gai Liang San Jia San (2:2:1:1:2:2) (Miao, 2018)	Honey-fried Tortoise Shell, Honey-fried Turtle Shell, Honey-fried Ground Beetle, Honey-fried Earthworm, Acorus Tatarinowii Rhizome, Prepared Polygonum Multiflora	LPS(5ug/mL)	BV2 cells PC12 cells	5% drug-containing CSF	Huperzine A	Drug-free CSF	24 h	*In vitro*	↑P62, P-mTOR, IL-4, IL-10 ↓LC3, Beclinl, PI3K, P-AKT, IL-1β, IL-6, TNF-α
Shen Rong He Ji (Yabin, 2018)	Ginseng, Poria, Cistanche, Processed Fo-ti, Alpinia Oxyphylla, Anemarrhena Rhizome, Acorus Tatarinowii Rhizome, Polygala Root, Szechuan Lovage Rhizome, Red Peony Root, Achyranthes Root	Aβ_25-35_(3uL) H.I.	KM mice	26, 13, 6.5 g/kg	Donepezil	Water	21 days	*In vivo*	↑P-PI3K, P-GSK-3β, P-AKT ↓P-Tau(Ser404,Thr181,Thr231,Ser396)
Jin Si Wei (GAPT) (Li, 2017)	Cistanche, Ginseng, Acorus Tatarinowii Rhizome, Curcuma Root, et al	—	APP/PS1	20, 10, 5 g/kg	Donepezil	0.5%CMC	3 months	*in vivo*	↑GLUT1, GLUT3, PI3K, AKT, P-GSK-3β ↓P-mTOR
Jin Si Wei (GAPT) (Zhuo, 2022)	Cistanche, Ginseng, Acorus Tatarinowii Rhizome, Curcuma Root, et al	—	APP/PS1	20, 10, 5 g/kg	Donepezil	0.5%CMC	8 months	*in vivo*	↑BDNF, PI3K, P-AKT, P-AKT/AKT, P-mTOR, P-mTOR/mTOR ↓P-Tau, AKT, mTOR
Bu Yuan Qing Nao Granules (5:5:4:5:4) (Yihong, 2015)	Acorus Tatarinowii Rhizome, Polygala Root, Ginseng, Poria, Prepared Rehmannia Root, et al	Aβ_25-35_(10ug) H.I.	Wistar rats	20, 10, 5 g/kg	Donepezil	0.9%Nacl	4 weeks	*in vivo*	↑PI3K, AKT, Bcl-2 ↓APP, Bax
Er Zhi Wan (3:2) (Xie et al., 2021)	Ligustrum Fruit, Eclipta	ovariectomy + D-gal(100 mg/kg) i.p +Aβ_1-40_(10ug) H.I.	SD rats	1.5, 0.75 g/kg	Estradiol valerate	0.9%Nacl	35 days	*In vivo*	↑AKT, PI3K, Bcl-xl, Bcl-2 ↓GSK-3β, Bad
Alpinia Oxyphylla (Li et al., 2022)	—	H_2_O_2_(90uM)	PC12 cells	10, 20, 40, 60, 80, 100 μg/mL	—	1640 culture medium	2 h	*In vitro*	↑Bcl-2, P-PI3K, P-AKT ↓Bax, caspase 3
Shen Qi Wan (8:4:4:3:3:3:1:1) (Huang et al., 2022a)	Rehmannia Root, Chinese Yam, Cornelian Cherry Fruit, Alisma Rhizome, Poria, Tree Peony Bark, Cinnamon Twig, Aconite Root	STZ(5uL) H.I.	SD rats	1.5, 3, 6 g/kg	—	0.9%Nacl	28 days	*In vivo*	↑Bcl-2, PI3K, PDK1, P-AKT, GSK-3β ↓Bax, caspase-3
Alpinia Oxyphylla-Schisandra Berry (Qi et al., 2020)	—	Aβ_1-42_(20uM)	PC12 cells	50, 100uM	—	Serum-free medium	20 h	*In vitro*	↑P-PI3K/PI3K, P-AKT/AKT, AKT, P-CREB/CREB ↓P-Tau
Lychee Seed (Sun et al., 2020)	—	Aβ_25-35_(10ug)H.I.	SD rats	120, 240, 480 mg/kg	Donepezil	0.9%Nacl	28 days	*In vivo*	↑AKT ↓P-Tau, GSK-3β
Silver Bupleurum (Long et al., 2023)	—	—	*C. elegans* strains	25, 50, 100, 200, 500ug/mL	—	0.5 M NaOH +1% NaClO	36 h	*In vitro*	↑P-PI3K/PI3K, P-AKT/AKT
Xing Nao Jing (Liu et al., 2020)	Natural Musk, Borneol, Gardenia Flower, Turmeric	Aβ_1-42_(4 mg) H.I.	C57BL/6 (B6)mice	2, 5 mL/kg	MEM	0.9%Nacl	31 days	*In vivo*	↑P-AKT, P-mTOR
Yuan-Zhi Decoction (1:2:2:2:2:2:4:4) (Wu et al., 2022)	Polygala Root, Ginseng, Acorus Tatarinowii Rhizome, Notopterygium Root, Wild Ginger, Ephedra, Red Peony Root, Atractylodes Macrocephala	—	APP/PS1 mice	0.11, 0.32, 0.96 g/kg	Donepezil	Water	3 months	*In vivo*	↑PI3K, P-AKT/AKT ↓P-GSK-3β/GSK-3β
Asafetida (Huang et al., 2022b)	—	Scopolamine(3 mg/kg) i.g H2O2(175 μmol/mL)	C57BL/6 mice PC12 cells	150, 75, 37.5 mg/kg 0.1, 1, 10 mg/mL	— —	Water Culture medium	14 days 24 h	*In vivo* *In vitro*	↑Bcl-2, PI3K, P-AKT, P-GSK-3β ↓Bax
Andrographis (Gu et al., 2020)	—	OKA(90 nM)	PC12 cells	0.625, 1.25 mg/mL	—	Culture medium	—	*In vitro*	↑P-GSK-3β ↓BACE, NF-κB, PTGS2
Fu Fang Dan Shen (Liang et al., 2018)	Salvia Miltiorrhiza Root, Notoginseng, Borneol	Aβ_1-42_(4uM) D-Gal(60 mg/kg) i.p. +Alcl3(10 mg/kg) i.p	SH-SY5Y cells balb/c mice	4, 20ug/mL 0.05, 4.20 mg/kg	— —	Serum-free medium 0.9%Nacl	1 h 14 days	*In* *vitro* *In vivo*	↑P-AKT ↓Bad
Qing Xin Kai Qiao Fang (2:2:2:2:2:2:2:1.5:1.5:1) (Lin et al., 2020)	Raw Rehmannia Root, White Peony Root, Ophiopogon Root, Tree Peony Bark, Poria, Dendrobium, Acorus Tatarinowii Rhizome, Anemarrhena Rhizome, Sophora Root, Dried Tangerine Peel	—	APP/PS1	19, 9.5, 4.75 mg/kg	Donepezil	0.9%Nacl	3 months	*In vivo*	↑P-PI3K, P-AKT ↓GSK-3α, A β
Galangal (Huang et al., 2019)	—	H_2_O_2_(200uM)	PC12 cells	20, 40, 80uM	—	Culture medium	24 h	*In vitro*	↑P-AKT,P-mTOR ↓P-Tau
Paper Mulberry Fruit (Li et al., 2020)	—	—	APP/PS1 MICE	0.02, 0.03, 0.06 g/kg	RAPA	Water	2 months	*In vivo*	↑AKT, β-catenin
Yi Zhi Fang Dai Decoction (Liu W. et al., 2016)	Ginkgo Leaf, Ginseng, Cistanche, Acorus Tatarinowii Rhizome	Aβ_1-42_(10uM)	SY5Y cells	50, 100ug/mL	—	Culture medium	2 h	*In vitro*	↑AKT, P-AKT ↓caspase 12, caspase 3
Jinsiwei(GAPT) (Lan et al., 2024)	Cistanche, Ginseng, Acorus Tatarinowii Rhizome, Curcuma Root, et al	Aβ_25-35_(20uM)	SY5Y cells	10% drug-contain serum	—	Culture medium	24 h	*In vitro*	↑PI3K ↓GSK-3β, P-Tau
Banxia Xiexin Decoction(3:2:1:2:2:2:3) (Gao et al., 2024)	Pinellia ternata, Radix scutellariae, Rhizoma coptidis, Rhizoma zingiberis, Radix ginseng, Radix Glycyrrhizae Preparata, Fructus jujubae	—	APP/PS1 mice	6 g/kg	liraglutide	0.5%CMC	3 months	*In vivo*	↑GLP-1R, AKT, PI3K, P- PI3K
Jiedu Yizhi formula(1:2:1:1:1:1:1) (Cui et al., 2024)	Coptidis Rhizoma, Alpiniae Oxyphyllae Fructus, Chuanxiong Rhizoma, Pheretima, Carapax et Plastrum Testudinis Colla, Corni Fructus, and Rhei Radix et Rhizoma	—	APP/PS1 mice	10, 20 g/kg	Donepezil	0.9%Nacl	8 weeks	*In vivo*	↑Bcl-2 ↓Bax, caspase-3
Tiaobu Xinshen Prescription(15:15:15:10:15:9:6:15:12:6:9) (Zhiying et al., 2024)	Processed Fo-ti Root, Polygonatum, Astragalus Root, Angelica Sinensis, Wolfberry, Schisandra Berry, Thinleaf Milkwort, Cornus Officinalis, Codonopsis Root, Acorus Tatarinowii, Paeonia Rubra	—	5×FAD mice	4.18 g/kg	Donepezil	0.9%Nacl	60 days	*In vivo*	↑Synaptophsin, PSD-95, P-NMDAR1/NMDAR1, P-CaMKⅡa/CaMKⅡa, PI3K, P-AKTAKT
Sijunzi Decoction (Yanjun et al., 2024)	Ginseng, White Atractylodes Rhizome, Poria, Licorice Root	—	APP/PS1 mice	2.5, 10 g/kg	—	Water	4 months	*In vivo*	↑SOD ↓AChE, MDA
Jiaotaiwan(10:1) (Yan et al., 2024)	Cinnamomum Cassia, Coptis Chinensis	—	APP/PS1 mice	2.1, 4.2 8.4 g/kg	Donepezil	0.5%CMC	4 weeks	*In vivo*	↑PI3K, AKT, InR, GLUT1, GLUT3, GLUT4 ↓Aβ_42_, GSK-3β
Liuwei Dihuang medicine(8 : 4: 4 : 3: 3 : 3) (Yuan et al., 2023)	Radix rehmannia, Fructus corni, Rhizoma dioscoreae, Rhizoma alismatis, Cortex moutan, and Poria cocos	—	APP/PS1 mice	10 mL/kg	Donepezil	0.9%Nacl	60 days	*In vivo*	↑PI3K, P-AKT/AKT ↓Aβ_1-42_, IL-10, IL-1β
Ginkgo biloba leaf (Zhu et al., 2024)	—	—	APP/PS1 mice	1.5, 3, 6 mL/kg	—	0.9%Nacl	4 weeks	*In vivo*	↑P-AKT, P-PI3K ↓P-NF-κB, IL-1β, TNF-α, IL-6
Guben-Jiannao Ye(15:12:15:12:15) (Mao et al., 2024)	Codonopsis pilosula (Franch.) Nannf., Wolfiporia cocos (Schw.) Ryv.&Gibn. Lycium barbarum L., Crataegus pinnatifida Bunge. Ziziphus jujuba Mill	—	APP/PS1 mice	8.97 g/kg	—	water	3 months	*In vivo*	↑PSD95, SYN1, GAP43, P-AKT P-PI3K,P-mTOR
Lancao decoction (Wu et al., 2025)	Peilan	—	APP/PS1 mice	2.5 g/kg	Donepezil	0.9%Nacl	14 days	*In vivo*	↑P-PI3K, P-AKT, P-ERK

Note: “↑” indicates that the traditional Chinese medicine upregulates the expression of this protein, while “↓” indicates that the traditional Chinese medicine downregulates the expression of this protein.i.p.,intraperitoneally administered; i.g.,intragastrically administered; s.c.,subcutaneously injected; H.I., Hippocampal injection.Pos C,positive control; Neg C,negative control.

**TABLE 2:** Active metabolites of traditional Chinese medicine that intervene in the PI3K/AKT pathway for the treatment of AD.

metabolites	botanical drug source	Experiments	Animal or cell	Dose range	Pos C	Neg C	Duration	Model	Molecular mechanisms and outcomes
Saururus Chinensis Extract (Ying-nan et al., 2019)	Angelica Dahurica (root)	—	APP/PS1 mice	20 mg/kg	—	0.5%CMC	6 weeks	*In vivo*	↑PI3K, P-AKT/AKT, P-GSK-3β/GSK-3β ↓P-Tau(Ser202)
Danshenone II A (Yi et al., 2021)	Salvia Miltiorrhiza(root and rhizome)	LPS(0.5 g/L)H.I.	ICR mice	1, 5, 10 mg/kg	—	0.9%Nacl	7 weeks	*In vivo*	↑BDNF, AchE, P-PI3K/PI3K, P-AKT/AKT ↓AchE, IL-6, TNF-α, IBA-1, GFAP, P-NF-κB/NF-κB, TLR4
Essential Oil of Warm Curcuma (Yue et al., 2017)	Curcuma (rhizome)	Aβ_25-35_(3uL) H.I.	KM mice	6, 18 mg/kg	Donepezil	water	15 days	*In vivo*	↑PI3K, AKT ↓P-Tau(Thr231)/P-Tau (Ser404)
Crocinonthe (Yan et al., 2020a)	Saffron (stigma)	Aβ_25-35_(10uM)	rat hippocampal neuron cells	1,3, 10uM	—	DMSO	24 h	*In vitro*	↑PI3K, AKT, Bcl-2, P-CREB ↓Bax
Hydroxy-α-Sanshool (Lan, 2021)	Sichuan Pepper (pericarp)	H_2_O_2_(90uM)	PC12 cells	60, 30, 15uM	—	RPMI 1640 medium	2 h	*In vitro*	↑P-AKT, P-PI3K, AKT, Bcl-2 ↓caspase 3, Bax
Isoliquiritin (Wang C. et al., 2020)	Forsythia (fruit)	L-Glu(25uM) —	HT22 cells APP/PS1 mice	20uM 2.5, 5, 10, 20 mg/kg	— —	Culture medium 0.9%Nacl	3 h 42 days	*In vitro* *In vivo* APP/PS1 AD mice	↑Bcl-2, Bcl-xL, P-PI3K, P-AKT ↓Bad, Bax, Bid, AIF
Panax notoginseng saponins (Xin, 2016)	Notoginseng (root and rhizome)	—	SAMP8 mice	100, 200 mg/kg	Huperzine A	0.9%Nacl	8 weeks	*In vivo*	↑PI3K, AKT, P-AKT
Deer Antler Peptide (Xin, 2021)	Deer Antler (immature antler)	Aβ_42_(10uM)	BV2 cells	2, 5, 10uM	—	Culture medium	24 h	*In vitro*	↑SOD, GSH-PX, PI3K, AKT ↓MDA, Bad, Bax, caspase 3
Curcumin (Chen, 2013)	Turmeric (rhizome)	—	APP/PS1 mice	1000, 160 mg/kg	—	water	6 months	*In vivo*	↑MAP-2, LC3Ⅰ/Ⅱ ↓PI3K, AKT, mTOR
Icariin (Taolin et al., 2016)	Epimedium (leaves)	Aβ_25-35_(20uM)	PC12 cells	20uM	—	Culture medium	1 h	*In vitro*	↑P-AKT, P-GSK-3β
Puerarin (Zheng-rong et al., 2016)	Pueraria (root)	—	APP/PS1 mice	40, 80 mg/kg	Huperzine A	0.9%CMC	3 months	*In vivo*	↑P-GSK3β(Ser9) ↓Aβ_1-40_, Aβ_1-42_, P-Tau(T231)
Aconitine (Zhi-quan et al., 2021)	Aconite (root or tuberous root)	Aβ_1-40_(20uM)	SY5Y cells	5 nM	—	Culture medium	12 h	*In vitro*	↓GSK-3β(Tyr216)
Polygala Saponin + β-Asarone (Junping et al., 2018)	Polygala (root),Asarum (whole herb or root)	Aβ_25-35_(1uM)	HT22 cells	5, 10, 20uM	—	Culture medium	30min	*In vitro*	↑AKT, GSK-3β ↓ROS
Ginsenoside CK (Jia-nan et al., 2021)	Ginseng (root)	Aβ_42_(10uM)	BV2 cells	2, 5, 10uM	—	Culture medium	24 h	*In vitro*	↑PI3K, AKT, P-AKT, mTOR
Acorus Volatile Oil + Total Ginsenosides (Zhen, 2016)	Acorus Tatarinowii (rhizome),Ginseng (root)	D-gal(150 mg/kg) i.g. +Alcl3(5 mg/kg) i.p	KM mice	10 g/kg, 30, 150 mg/kg	Donepezil	Water	40 days	*In vitro*	↑ChAT, P-AKT, P-mTOR, APP, Beclin-1 ↓Aβ_1-42_, AChE
Lychee Seed Polyphenols (Xiong et al., 2020)	Lychee (fruit)	DXM(0.03125-2 uM)	HepG2 and HT22 cells	10ug/mL	—	Culture medium	48 h	*In vitro*	↑P-PI3K(Tyr199/Tyr458), P-AKT(Thr308), P-GSK-3β(Ser9) ↓P-Tau(Ser404)
Derivatives of Evodia Alkaloids (Pang et al., 2023)	Evodia (ripe)	APP/PS1 mice, 3 × Tg mice SH-SY5Y, HepG2 cells	20,200ug/kg 1,0.1, 0.01ug/mL	— —	Water Culture medium	4 weeks 24 h		*In vivo* *In vitro*	↑P-PI3K, P-AKT, P-GSK-3β(Ser9) ↓P-Tau(Thr181)
Schisandrin + Norcoclaurine (Qi et al., 2020)	Schisandra (fruit), Alpinia Oxyphylla (ripe fruit)	Aβ_1-42_(20uM)	PC12 cells	0.31, 0.62, 1.25, 2.5,5, 10, 20uM/1.56, 3,12, 6.25, 12.5, 25, 50, 100uM	—	Culture medium	4 h	*In vitro*	↑P-PI3K/PI3K, P-AKT/AKT, P-GSK-3β/GSK-3β, P-mTOR/mTOR, Bcl-2 ↓IL-1β, IL-6,TNF-α, caspase 3
Schisandrin (Zhao et al., 2019)	Schisandra (fruit)	Aβ_1-42_(10uM)	SY5Y cells	10uM	—	Culture medium	24 h	*In vitro*	↑P-AKT, P-GSK-3β ↓P-Tau
Flavonoids from the Stems and Leaves of Scutellaria baicalensis (Liu et al., 2022)	Scutellaria baicalensis (root)	Aβ_25-35_+AlCl3+RHTGF-β1	SD rats	35, 70, 140 mg/kg	—	Water	43 days	*In vivo*	↑P-AKT/AKT, PI3K
Astragaloside (Yang et al., 2023)	Astragalus (root)	Aβ_25-35_(20uM) —	HT22 cells APP/PS1 mice	0.625, 1.25, 2.5, 5, 10, 20, 40, 80uM 10, 20,40 mg/kg	— —	Culture medium 0.09%Nacl	4 h 1 months	*In vitro* *In vivo*	↑P-PI3K/PI3K, P-AKT/AKT, P-mTOR/mTOR
Paeoniflorin (Ma et al., 2018)	Peony (root)	OA(40 nM)	SH-SY5Y cells	50, 100, 200uM	—	Culture medium	8 h	*In vitro*	↑P-GSK-3β(Ser9), P-AKT(Ser473) ↓P-Tau(Ser404)
Rg1 (Fang et al., 2025)	Ginsenoside(root)	Aβ_1-42_(10uM)	BV2 cells	10uM	—	Culture medium	24 h	*In vitro*	↑LC3B II/I, Bcl-2, P-PI3K/PI3K, P-AKT/AKT
Crocin (Wang S. et al., 2024)	Saffron(stigmas)	Aβ_25-35_(2uL)H.I.	ICR mice	40 mg/kg	—	0.9%Nacl	14 days	*In vivo*	↑P-PI3K, P-AKT
Lycium Barbarum Polysaccharides (Yang et al., 2024)	Lycium barbarum(fruits)	STZ(3 mg/kg)H.I.	C57BL/6J mice	50, 100. 200 mg/kg	Donepezil	0.9%Nacl	28 days	*In vivo*	↑P-GSK-3β, P-IRS1/IRS1, P-PI3K/PI3K ↓P-Tau(Thr205, Ser396, Thr205
Curcumin (Yang et al., 2024)	Curcuma longa(rhizomes)	Aβ_1-42_(2uL)H.I.	C57BL/6J mice	100 mg/kg	—	0.9%Nacl	14 days	*In vivo*	↑β-catenin, BDNF, P-AKT, P-GSK-3β,P-CREB
Hyperoside (Wang et al., 2023)	Acanthopanax senticosus Hypericum(leaves or flowers)	Aβ_42_(2.5uM)	PC12 cells	10, 20, 30uM	—	Culture medium	24 h	*In vitro*	↑P-PI3K, P-AKT, Nrf2, HO-1
Icariside Ⅱ (Yijun et al., 2024)	Epimedium(aerial)	—	APP/PS1 mice	10 mg/kg	Donepezil	0.5%CMC	30 days	*In vivo*	↑PI3K, P-AKT/AKT ↓caspase 3, Bax/Bcl-2
Dendrobium nobile Lindl. alkaloid (Hu et al., 2024)	Dendrobium nobile Lindl(stems)	wortmannin + GF-109203X (5uM) wortmannin (5uL)+GF-109203X (5uL)H.I.	N2a cells Wistar rats	0.35, 3.5, 35 ng/mL 20, 40 mg/kg	— Donepezil	Culture medium 0.9%Nacl	24 h 2 weeks	*In vitro* *In vivo*	↑P-GSK-3β, P-AKT ↓P-Tau

Note: “↑” indicates that the traditional Chinese medicine upregulates the expression of this protein, while “↓” indicates that the traditional Chinese medicine downregulates the expression of this protein. i.p.,intraperitoneally administered; i.g.,intragastrically administered; s.c.,subcutaneously injected; H.I., Hippocampal injection.Pos C,positive control; Neg C,negative control.

**TABLE 3:** Anti-AD active ingredients of Chinese botanical drug.

Classifications	Type of extract	Botanical drug	Family
Carotenoids	Crocin	Extracted from the stigmas of *Crocus sativus* (Saffron) [Iridaceae; Saffron Stigma]	Iridaceae
Polypeptides	Deer Antler Peptide	Extracted from the immature antlers of *Cervus elaphus* or *Cervus nippon* [Cervidae; Cervi Cornu Pantotrichum]	Cervidae
Polyphenols	Curcumin	Extracted from the rhizomes of *Curcuma longa* (Turmeric) [Zingiberaceae; Curcumae Longae Rhizoma]	Zingiberaceae
Flavonoids	Curcumin	Extracted from the rhizomes of *Curcuma longa* [Zingiberaceae; Curcumae Rhizoma]	Zingiberaceae
Flavonoids	Icariin	Extracted from the leaves of *Epimedium brevicornu* Maxim. [Berberidaceae; Epimedii Folium]	Berberidaceae
Flavonoids	Puerarin	Extracted from the roots of *Pueraria lobata* (Willd.) Ohwi [Fabaceae; Puerariae Radix]	Fabaceae
Flavonoids	Flavonoids from the Stems and Leaves of Scutellaria baicalensis	Extracted from the dried stems and leaves of *Scutellaria baicalensis* Georgi [Lamiaceae; Scutellariae Radix]	Lamiaceae
Flavonoids	Isoliquiritin	Extracted from the roots of *Glycyrrhiza uralensis* Fisch. [Fabaceae; Glycyrrhizae Radix]	Fabaceae
Flavonoids	Crocinonthe	Extracted from the dried stigmas of *Crocus sativus* L. [Iridaceae; Croci Stigma]	Iridaceae
Flavonoids	Hyperoside	Extracted from the leaves or flowers of plants such as *Crataegus pinnatifida* (Hawthorn) [Rosaceae]	Rosaceae
Flavonoids	Icariside II	Extracted from the aerial parts of *Epimedium* species (commonly *Epimedium brevicornum*) [Berberidaceae]	Berberidaceae
Alkaloids	Aconitine	Extracted from the processed lateral roots of *Aconitum carmichaelii* Debeaux [Ranunculaceae; Aconiti Lateralis Radix Praeparata]	Ranunculaceae
Alkaloids	Dendrobium nobile Lindl. alkaloid	Extracted from the stems of *Dendrobium nobile Lindl.* (Noble Dendrobium) [Orchidaceae; Dendrobii Caulis]	Orchidaceae
Saponins	Polygala Saponin	Extracted from the roots of *Polygala tenuifolia* Willd. [Polygalaceae; Polygalae Radix]	Polygalaceae
Saponins	Panax notoginseng saponins	Extracted from the roots of *Panax notoginseng* (Burk.) F.H.Chen [Araliaceae; Notoginseng Radix et Rhizoma]	Araliaceae
Essential Oils	Acorus Volatile Oil	Extracted from the rhizomes of *Acorus calamus* L. [Acoraceae; Acori Tatarinowii Rhizoma]	Acoraceae
Essential Oils	Essential Oil of Warm Curcuma	Extracted from the rhizomes of *Curcuma longa* L. [Zingiberaceae; Curcumae Rhizoma]	Zingiberaceae
Phenols	Lychee Seed Polyphenols	Extracted from the seeds of *Litchi chinensis* Sonn. [Sapindaceae; Litchi Semen]	Sapindaceae
Alkaloids	Derivatives of Evodia Alkaloids	Extracted from the fruits of *Evodia rutaecarpa* (Juss.) Benth. [Rutaceae; Evodiae Fructus]	Rutaceae
Alkaloids	Norcoclaurine	Extracted from the rhizomes of *Coptis chinensis* Franch. [Ranunculaceae; Coptidis Rhizoma]	Ranunculaceae
Lignans	Schisandrin	Extracted from the dried fruits of *Schisandra chinensis* (Turcz.) Baill. [Schisandraceae; Schisandrae Chinensis Fructus]	Schisandraceae
Quinones	Danshenone II A	Extracted from the roots of *Salvia miltiorrhiza* Bunge [Lamiaceae; Salviae Miltiorrhizae Radix et Rhizoma]	Lamiaceae
Saponins	Total Ginsenosides	Extracted from the roots of *Panax ginseng* C.A.Mey. [Araliaceae; Ginseng Radix et Rhizoma]	Araliaceae
Saponins	Ginsenoside CK	Extracted from the roots of *Panax ginseng* C.A.Mey. [Araliaceae; Ginseng Radix et Rhizoma]	Araliaceae
Saponins	Ginsenoside 1	Extracted from the roots of *Panax ginseng* C.A.Mey. [Araliaceae; Ginseng Radix et Rhizoma]	Araliaceae
Saponins	Astragaloside	Extracted from the roots of *Astragalus membranaceus* (Fisch.) Bunge [Fabaceae; Astragali Radix]	Fabaceae
Polysaccharides	Lycium Barbarum Polysaccharides	Extracted from the fruits of *Lycium barbarum* (Goji Berry) [Solanaceae; Lycii Fructus]	Solanaceae
Other Active Components	Paeoniflorin	Extracted from the roots of *Paeonia lactiflora* Pall. [Paeoniaceae; Paeoniae Radix Alba]	Paeoniaceae
Other Active Components	Hydroxy-α-Sanshool	Extracted from the dried pericarps of *Zanthoxylum bungeanum* Maxim. [Rutaceae; Zanthoxyli Pericarpium]	Rutaceae
Other Active Components	β-Asarone	Extracted from the rhizomes of *Acorus calamus* L. [Acoraceae; Acori Tatarinowii Rhizoma]	Acoraceae
Other Active Components	Saururus Chinensis Extract	Extracted from the whole dried plant of *Saururus chinensis* (Lour.) Baill. [Saururaceae; Saururi Herba]	Saururaceae

Although herbal formulations and extracts derived from TCM have demonstrated great potential in regulating the PI3K/AKT pathway for treating AD, corresponding clinical studies are still lacking, particularly those on individual Chinese herbal compounds. Additionally, in related experimental studies, some lack positive control drugs, some have undefined compositions or ratios of herbal formulas (with their components not yet disclosed), and others rely solely on *in vitro* experiments. More comprehensive *in vivo* studies are needed to simulate the complex environment within organisms, explore their mechanisms of action, and assess the efficacy and safety of long-term use of TCM components. The study of herbal formulas faces numerous challenges, such as the complexity of TCM components making it difficult to elucidate their mechanisms, as well as variability in sourcing, species, processing methods, and formulations that can affect reproducibility. In the future, standardization of formula preparation and use will need further development. Employing analytical approaches such as metabolomics and network pharmacology could facilitate higher-quality, more in-depth research.

Specific components of TCM regulate the PI3K/AKT signaling pathway through multiple levels and targets, either directly or indirectly. This regulation involves activating upstream receptors, modulating key enzymes, and influencing downstream effector proteins (Kumar and Bansal, 2022). For example, icariin can enhance the expression of IGF and BDNF, thereby activating the PI3K/AKT pathway, inhibiting Aβ production and tau protein phosphorylation, and alleviating symptoms in AD animal models (Wang et al., 2012). Curcumin improves adult neurogenesis in AD mice by increasing BDNF expression and activating the PI3K/AKT pathway (Lou et al., 2024). The herbal formula Jin Siwei(GAPT) boosts glucose uptake in specific brain regions of AD mice, increases glucose transport, and repairs damaged PI3K/AKT signaling pathways (Li, 2017). Ginsenoside CK upregulates PI3K, AKT, and phosphorylated AKT in AD model cells (Jia-nan et al., 2021). Herbal formulation Erzhi Pill enhances the expression of Akt and PI3K, participating in the regulation of key enzymes within the PI3K/AKT pathway (Xie et al., 2021). Herbal formulation Xingnaojing lowers GSK-3β expression and raises mTOR expression, exerting regulatory effects through downstream effector proteins of the PI3K/AKT pathway (Liu et al., 2020). Although existing studies have uncovered how certain TCM components influence the PI3K/AKT pathway, the direct targets and specific regulatory mechanisms of some components remain unclear. Further investigation is warranted in future research.

In addition to the PI3K/AKT pathway, research has shown that herbal formulations and extracts derived from TCM can improve AD pathology through multiple targets by modulating pathways such as NF-κB, Nrf2, JAK/STAT, ubiquitin-proteasome, PPARα, AMPK/mTOR, and SIRT1 (Ding et al., 2022). For instance, Naoxintong capsule has been found to improve cognitive function in APP/PS1 mice by downregulating the expression of IL-1β, IL-6, TNF-α, NF-κB, Aβ, and phosphorylated Tau (p-Tau) (Wang et al., 2021). Qin et al. demonstrated that astragalus polysaccharide increases nuclear Nrf2 expression as well as SOD and GSH-Px activity, reduces MDA accumulation, alleviates oxidative stress damage, and enhances spatial learning and memory in APP/PS1 mice (Qin et al., 2020). Long et al. reported that Suanzaoren decoction alleviated cognitive deficits in an AD mouse model, reduced Aβ plaque deposition and neuronal loss, downregulated the expression of p-JAK2-Tyr1007 and p-STAT3-Tyr705 proteins, and modulated the JAK2/STAT3 pathway (Long et al., 2021). Another study found that protopine, a component of Corydalis, exhibited neuroprotective effects in P301S Tau and 3xTg-AD mouse models by inhibiting histone deacetylase 6 activity while enhancing the expression of molecular chaperones such as HSP70, HSP90, HSC70, and acetylated HSP90, thereby influencing the ubiquitin-proteasome pathway in AD (Sreenivasmurthy et al., 2022).

At present, most basic studies on the intervention of AD with TCM are conducted using animal or cell models. Animal models can realistically simulate the pathological features of AD in living organisms, while cell models allow researchers to investigate the disease mechanisms at a microscopic level. However, existing AD models have certain limitations. Different cell models can only represent certain aspects of AD pathogenesis and fail to replicate its full complexity. The criteria for establishing cell models are not yet standardized; using changes in cell viability or the expression of certain factors as evaluation metrics cannot fully reflect the pathological features of AD. Moreover, the effects of various metabolic intermediates, ions, serum components, and substrates on cell growth and differentiation during cell culture require further exploration (Ting et al., 2020). Similarly, animal models can only partially mimic the pathological characteristics and clinical symptoms of AD and cannot comprehensively represent all the pathological, biochemical, and neurobehavioral changes. Therefore, screening drugs with one or two AD cell or animal models, or using a combined modeling approach, may be more convincing than relying on a single model (Lei and Xiaoli, 2020).

Currently, drugs directly targeting the PI3K/AKT pathway in AD treatment remain in the research stage. Exploring herbal formulations and extracts derived from TCM modulates the PI3K/AKT pathway in treating AD could provide valuable insights for developing synthetic drugs targeting this pathway. TCM contains numerous active compounds that can exert synergistic effects, intervening at multiple targets and regulating both upstream and downstream molecules of the PI3K/AKT pathway. In the future, the development of multi-target drugs may be necessary to address the complex pathological mechanisms of AD.

Many herbal formulations and extracts derived from TCM extracts have demonstrated promising therapeutic effects against AD. It is worth considering whether derivatives based on these extracts could be designed to offer greater potency, stability, and selectivity as PI3K/AKT-targeting agents. For example, curcumin is quickly eliminated in the body due to its hydrophobicity and low bioavailability (Mirzael et al., 2017). Structural modifications of curcumin, synthesis of a series of derivatives, or the use of nanoemulsions to improve oral bioavailability might address these issues (Ghasemi et al., 2020; Liu L. et al., 2016). As our understanding of the role of herbal formulations and extracts derived from TCM in modulating this pathway and its involvement in AD pathogenesis deepens, new therapeutic strategies may emerge.

Currently, treatment for AD primarily includes cholinesterase inhibitors, NMDA receptor antagonists, and monoclonal antibody-based drugs. These therapies mainly target short-term cognitive improvements and slow disease progression, but they are associated with varying degrees of adverse effects and have limited efficacy for moderate to severe AD patients. In contrast, numerous herbal formulations and extracts derived from TCM have shown promising therapeutic effects in basic experimental studies. Some TCM formulations have also been tested in clinical research with encouraging results. A multicenter, randomized, observer-blind controlled trial confirmed that modified Guipi Decoction significantly improved BPSD in AD patients (Nogami et al., 2023). Wang HC et al. found that Jiannao Yizhi Formula was as effective and safe as donepezil for treating mild to moderate AD (Wang H. C. et al., 2020). Shenmai or Shenfu Injection and Huannao Yicong Formula have been demonstrated to be effective and safe for treating mild to moderate AD (Chen et al., 2015; Zhang et al., 2015). Liu P et al. reported that treatment with Reinforcing Kidney Essence improved cognitive function and daily living in AD patients (Liu et al., 2013). Yu L et al. discovered that syndrome-differentiation-based TCM treatment effectively enhanced cognitive function in mild to moderate AD patients and improved brain connectivity between the posterior cingulate cortex and specific brain regions (Yu et al., 2012).

Clinical studies suggest that herbal formulations and extracts derived from TCM offer substantial potential for treating AD, with advantages such as fewer side effects, convenient administration, and lower treatment costs. However, clinical evaluations of TCM efficacy often rely on subjective symptom improvement rather than objective biomarker validation. Furthermore, TCM treatments lack standardized dosing and protocols, and some medications may have potential toxicity with long-term use. Therefore, high-quality, large-scale, multicenter clinical trials are still needed to verify the safety and efficacy of TCM in AD treatment.

## Glossary

AD: Alzheimer's disease
mTOR: Mammalian target of rapamycin
APP: Amyloid precursor protein
mTORC2: Mammalian target rapamycin complex 2
AKT: Protein kinase B
MDA: Malondialdehyde
Aβ: Amyloid-β
Nrf2: Nuclear factor erythroid 2-related factor 2
Bcl-XL: B cell lymphoma-extra large
NFT: Neurofibrillary tangles
Bcl-2: B-cell lymphoma-2
NF-κB: Nuclear factor kappa-b
Bax: Bcl-2-associated x protein
NS-PTEN KO: Neuron subgroup-specific pten knockout
BDNF: Brain-derived neurotrophic factor
PIP3: Phosphatidylinositol-3,4,5-bisphosphate
caspases: Cysteine-aspartate proteases
PIP2: Phosphatidylinositol-4,5-bisphosphate
CDK-5: Cyclin-dependent kinases
PI3K: Phosphoinositide 3-kinase
CNS: Central nervous system
PI3K/AKT: Phosphoinositide 3-kinase/protein kinase b
eNOS: Endothelial nitric oxide synthase
P-AKT: Phosphorylated akt
GSH: Glutathione
PH: Pleckstrin homology
GSK-3β: Glycogen synthase kinase-3β
PDK1: 3-phosphoinositide-dependent protein kinase-1
IR: Insulin receptor
ROS: Reactive oxygen species
IL-6: Interleukin-6
SOD: Superoxide dismutase
IRS: Insulin receptor substrates
TCM: Traditional chinese medicine
IKK: Inhibitor of kappa b kinase
TNF-α: Tumor necrosis factor-α
IL-1β: Interleukin-1β
ULK1: Unc-51-like kinase 1
